# A combination of yeast beta-glucan and milk hydrolysate is a suitable alternative to zinc oxide in the race to alleviate post-weaning diarrhoea in piglets

**DOI:** 10.1038/s41598-018-37004-9

**Published:** 2019-01-24

**Authors:** Anindya Mukhopadhya, John V. O’Doherty, Torres Sweeney

**Affiliations:** 1 School of Veterinary Medicine, University College Dublin, Dublin, Ireland; 2 School of Agriculture and Food Science, University College Dublin, Dublin, Ireland

## Abstract

Zinc oxide (ZnO) is currently used as a dietary supplement to support gut homeostasis during the standard ‘abrupt’ weaning practices in commercial pig production. However, a replacement is urgently required as a ban on ZnO usage is imminent. The objective of this study was to explore the potential of a bovine casein hydrolysate (5kDaR) and yeast β-glucan, and their combination, as an alternative to ZnO. Eighty 21d old male piglets received a basal diet or supplemented with 5kDaR and yeast β-glucan alone or in combination, or ZnO from the day of weaning and were monitored for 10 days (n = 8/group; dietary groups: control diet; control diet + 5kDaR; control diet + yeast β-glucan; control diet + 5kDaR + yeast β-glucan; control diet + ZnO). Individually, supplement yeast β-glucan or 5kDaR did not improve gut health. In contrast, the yeast β-glucan + 5kDaR combination supplement supported a healthy gut, indicated by healthy faecal scores and improved growth parameters; similar to ZnO inclusion (P > 0.05). There was no negative effect on the gut microbiota with yeast β-glucan + 5kDaR supplementation; while ZnO negatively affected the *Bifidobacterium spp*. abundance (P < 0.05). The inflammatory NFκB pathway was suppressed by yeast β-glucan + 5kDaR supplementation, similar to ZnO (P > 0.05). In conclusion, the dietary supplement yeast β-glucan + 5kDaR restored homeostasis of the newly weaned piglet gut similar to the widely used ZnO, and can potentially replace ZnO.

## Introduction

Weaning is typically initiated between 14–28 days after birth and is a critical period in commercial pig production systems. During this period, piglets are vulnerable to a variety of physiological and environmental stressors, as reviewed by Lallès *et al*.^1^. The ‘abrupt weaning’ that occurs in commercial pig production can result in reduced feed intake, with consequent effects on weight gain and both transitory and long-lasting modifications to the absorptive, secretory and barrier properties of the intestine^2^. There are also changes to the microbiota, characterised by a marked decrease in biodiversity^3^; which increases the risk of pathogenic bacteria such as enterotoxigenic *Escherichia coli* (ETEC) gaining a niche in the gastrointestinal tract (GIT). This often leads to post-weaning diarrhoea in the piglets^4,5^. Traditionally, the dietary inclusion of antibiotic growth promoters (AGP) and/or high concentrations of zinc oxide (ZnO) have been used to overcome the anticipated gastrointestinal disturbances at this vulnerable time^6–10^. Both the EU ban on AGPs and environmental concerns over the use of heavy metals have been highlighted by Sales^9^; driving efforts to identify natural bioactives with growth promoting and/or immunomodulatory properties which could be used to overcome these post-weaning complications^11–13^.

The process of identifying natural sustainable sources of bioactives that could support a healthy gastrointestinal system in both animals and humans include preliminary screening and testing of the compounds *in-vitro*. Milk hydrolysates, based on their source and chemical composition have wide ranging bioactive properties including anti-microbial and anti-inflammatory properties^14^. Yeast β-glucan^15^ also has bioactive properties across a range of different contexts. Beta-glucans are complex polysaccharides derived from a range of sources including plants, yeast and fungi with a variety of *in-vitro* bioactivities including anti-cancer, anti-inflammatory and immune-modulating properties, reviewed by Zhu *et al*.^16^. While the *in-vitro* screening approach is cost effective and non-invasive relative to *in-vivo* studies, many compounds reported to have bioactive properties *in-vitro* have reduced bioavailability in *in-vivo*; this is in part attributable to the exposure of the bioactive ingredient to digestive enzymes and alterations in gastric pH that subsequently alters the bioavailability of the compound^17^. In a previous study, a 5 kDa retentate (5kDaR), derived from moderate degree of hydrolysis (11–16%) of bovine milk casein, demonstrated potent anti-inflammatory activity both *in-vitro* and *ex-vivo* colonic models^18^. However, the anti-inflammatory activity of this 5kDaR protein hydrolysate was no longer evident when it was used as a feed supplement in an experimental weaning piglet model^19^.

Protecting the bioactive properties of any natural compound *in-vivo* is a growing area of interest^20^. Natural encapsulating and delivery agents like β-glucan, can be used to protect the digestion of bioactive compounds in the stomach^21,22^. Yeast β-glucans are of interest, as apart from serving as a natural delivery agent, they also elicit immunomodulatory effects, both *in-vitro* and *in-vivo*^15,23^. Hence, the objectives of this experiment were firstly to compare the effects of a milk protein hydrolysate (5kDaR), a yeast β-glucan and a combination of the 5kDaR + yeast β-glucan on growth performance and gut health in the newly weaned pig. Secondly, to determine if any of these supplements are comparable to ZnO. The hypothesis of this study was that a combination of yeast β-glucan and milk hydrolysate will support growth and gut health parameters similar to ZnO in a post-weaned pig diet.

## Materials and Methods

All procedures described in this experiment were conducted under the experimental license from the Irish Department of Health (License number: AREC-P-12-54-Sweeney; License holder: Prof. Torres Sweeney), in accordance with the Cruelty to Animals Act 1876 and the European Communities (amendments of the Cruelty to Animals Act 1876) regulations, 1994.

### Experimental Design and Dietary Treatments

This study was designed as a complete randomised design comprising of 5 dietary treatments. The dietary treatments were as follows: 1) control diet, 2) control diet + 5kDaR, 3) control diet + yeast β-glucan, 4) control diet + 5kDaR + yeast β-glucan and 5) control diet + ZnO (3.2 g/kg feed, pharmacological dose). The generation of 5kDaR, used in this study, from bovine milk derived sodium caseinate, has been reported previously^18^. The optimum concentrations of 5kDaR and yeast β-glucan were established from previous studies^19,23^. The diets were formulated to contain similar concentrations of digestible energy (DE) (14.5 MJ/kg) and standardized ileal digestible (SID) lysine (12.5 g/kg) contents. All amino acid requirements were met relative to SID lysine^24^. All diets were milled on site and fed in meal form for 10 days post-weaning. The ingredient composition and compositional analysis of the experimental diets are presented in Table 1.

**Table 1:** Ingredient and compositional analysis of the diets.

Ingredient (g/kg)	Control	5kDaR	yeast β-glucan	5kDaR + yeast β-glucan	ZnO
Whey powder	50	50	50	50	50
Wheat	380	380	380	380	376.8
Barley	233.5	233.25	233.25	233.0	233.5
Soya bean meal	170	170	170	170	170
Full fat soybean	120	120	120	120	120
Soya oil	10	10	10	10	10
Vitamins and minerals	3	3	3	3	3
Salt	3	3	3	3	3
Dicalcium phosphate	12.5	12.5	12.5	12.5	12.5
Limestone	11	11	11	11	11
Lysine HCL	4	4	4	4	4
DL-methionine	1.5	1.5	1.5	1.5	1.5
L-threonine	1.5	1.5	1.5	1.5	1.5
ZnO	0	0	0	0	3.2
5kDaR	0	0.25	0	0.25	0
β-glucan	0	0	0.25	0.25	0
**Chemical analysis**					
Dry matter	866.1	866.1	866.3	866.4	866.3
Crude protein (N × 6.25)	210.6	210.5	210.5	210.6	210.5
Digestible energy (MJ/kg)*	14.5	14.3	14.4	14.5	14.4
Ash	48.4	48.5	48.3	48.2	48.3
Neutral detergent fiber	115.1	114.0	113.1	114.1	115.6
Lysine*	14.5	14.5	14.6	14.5	14.6
Methionine and cysteine*	8.4	8.2	8.2	8.5	8.4
Threonine*	9.1	9.2	9.2	9.1	9.1
Tryptophan*	2.5	2.4	2.5	2.5	2.6
Calcium*	9.5	9.5	9.5	9.4	9.4
Phophorus*	6.1	6.2	6.0	6.1	6.1

*Calculated for tabulated nutritional composition^51^

Provided (mg/kg complete diet): Cu, 100; Fe, 140; Mn, 47; Zn, 120; I, 0·6; Se, 0·3; retinol, 1·8; cholecalciferol, 0·025; α-tocopherol, 67; phytylmenaquinone, 4; cyanocobalamin, 0·01; riboflavin, 2; nicotinic acid, 12; pantothenic acid, 10; choline chloride, 250; thiamin, 2; pyridoxine, 0·015. Celite included at 300 mg/kg complete diet.

### Animals and Management

Eighty male weaned piglets, twenty one days old, [progeny of Landrace boars x (Large White x Landrace) sows] were weighed and blocked on the basis of initial live weight (7.3 ± 0.2 kg) and assigned to one of the five dietary treatment groups and housed in pairs on fully slatted floors (1.68 × 1.22 m) (n = 8). Piglets were weighed individually at the beginning of the experiment (day 0 = day of weaning) and subsequently on days 5 and 10. The ambient environmental temperature within the house was thermostatically controlled at 30 °C and humidity was maintained at 65%. All feed and water was available *ad libitum*. Feed, in meal form, was available up to the final weighing and all remaining feed was re-weighed for the purposes of calculating average daily feed intake (ADFI) and gain to feed ratio (G:F).

### Faecal scoring

One measure of gut health in a live animal is the faecal score, which is performed by scoring the consistency of the faeces, as described previously^25^. From day 0 until day 10, faecal scores were assessed twice daily for each individual pen to indicate the presence and severity of diarrhoea. The following scoring system was used to assign faecal scores: 1 = hard, 2 = slightly soft, 3 = soft, partially formed, 4 = loose, semi-liquid, 5 = watery, mucous-like.

### Animal sacrifice and sample collection

On day 10, piglets were sacrificed following a lethal injection of Euthatal -pentobarbitone sodium BP- (Merial Animal Ltd, Sandringham House, Essex, UK) at a rate of 1 mL/1·4 kg live body weight. Following this, the digestive tract was surgically removed and tissue samples from duodenum, ileum and colon were collected in sterile phosphate buffer saline (PBS) (Oxoid, Basingstoke, UK). Digesta samples were then recovered aseptically from the caecum and colon and transported on ice for storage at −20 °C.

### Extraction of microbial DNA from caecal and colonic digesta

Samples of digesta (approximately 10 g) were recovered from the caecum and colon and stored in sterile 50 ml tubes (Sarstaedt, Wexford, Ireland) at −20 °C and transported to the laboratory within 2 h. Bacterial genomic DNA was extracted from digesta samples using the QIAamp DNA stool kit (Qiagen, West Sussex, UK) following manufacturer’s instructions. The quantity and quality of DNA was assessed using the Nanodrop spectrophotometer (Nanodrop, ND1000; Thermo Scientific, Wilmington, DE) and the purified DNA stored at −20 °C

### Microbial quantification using quantitative real time polymerase chain reaction (qPCR)

#### Standard curve preparation

A pooled aliquot of bacterial DNA derived from all animals was used to prepare standard curves for qPCR. Phylum, family, genus and strain specific primers were used to amplify the 16s rRNA gene (Table 2). Standard curves were subsequently generated by carrying out qPCR on serial dilutions of amplicons using the same genus and species-specific primers to permit absolute quantification, based on gene copy number^26^.

**Table 2:** Oligonucleotide sequence of forward and reverse primers used for qPCR of bacterial 16s rRNA.

Target bacterial groups	Forward primer (5′-3′) Reverse primer (5′-3′)	Amplicon size (bp)	Tm (°C)
Total bacteria	GTGCCAGCMGCCGCGGTAA GACTACCAGGGTATCTAAT	291	57
*Bacteroides*	AACGCTAGCTACAGGCTT CAAATGTGGGGGACCTTC	276	54
*Firmicutes*	GGAGYATGTGGTTTAATTCGAAGCA AGCTGACGACAACCATGCAC	126	59
*Bifidobacterium spp*.	CGG GTG AGT AAT GCG TGA CC TGA TAG GAC GCG ACC CCA	125	59
*Lactobacillus spp*.	TGGATCACCTCCTTTCTAAGGAAT TGTTCTCGGTTTCATTATGAAAAAATA	340	55
*Enterobacteriaceae*	CATTGACGTTACCCGCAGAAGAAGC CTCTACGAGACTCAAGCTTGC	190	58
AEEC strain	GGCGATTACGCGAAAGATAC GATTAACCTCTGCCGTTCCA	100	58

#### Estimation of selected bacterial groups in the caecal and colonic digesta

*Estimation of selected bacterial groups in the caecal and colonic digesta*: All reactions were performed on the ABI 7500 Fast PCR System (Applied Biosystems Ltd., Warrington, UK). For bacterial groups, qPCR was performed in a final reaction volume of 20 μL containing 1 μL template DNA, 1 μL of forward and reverse primers (25 pM), 10 μL Fast SYBR Green Master Mix (Applied Biosystems) and 8 μL nuclease-free water. The cycling conditions included a denaturation step of 95 °C for 10 min followed by 40 cycles of 95 °C for 15 s and 65 °C for 1 min. All reactions were performed in triplicate. Dissociation curves were generated to confirm the specificity of the resulting PCR products. The dry matter (DM) of the digesta was determined after drying overnight at 103 °C. Estimates of gene copy numbers for select bacteria were log transformed and are presented as gene copy numbers per gram of DM of digesta.

### Gene expression profiling using qPCR

#### RNA extraction

Tissue samples were collected from the duodenum, ileum and colon, rinsed with ice-cold sterile PBS (Oxoid, Basingstoke, UK) and then the overlying smooth muscle was stripped off. The samples were then cut into 1 cm^2^ sections using a sterile scalpel and stored in 5 ml of RNAlater™ (Applied Biosystems, Foster City, CA, USA) overnight, followed by storage at −20 °C prior to RNA extraction. Approximately 50 mg of tissue was used for RNA extraction, using the GenElute™ Mammalian Total RNA Miniprep Kit (Sigma–Aldrich Ireland Ltd., Co. Wicklow, Ireland), following the manufacturer’s instructions. Quantification of the total RNA was performed using 1.5 μL of total RNA on a NanoDrop™ Spectrophotometer ND1000 (Thermo Scientific, Wilmington, DE, USA) and samples with 260:280 ratio ≥2.0 were considered suitable for complementary DNA (cDNA) synthesis. Total RNA integrity (i.e. quality and quantity) was assessed by analysing 1 μl of total RNA using the Agilent 2100 Bioanalyser™ version A.02.12 (Agilent Technologies, Santa Clara, CA, USA) using RNA Nano LabChips® (Caliper Technologies Corporation, Mountain View, CA, USA).

#### cDNA synthesis

Total RNA (1 µg) was used for the synthesis of First Strand cDNA using the First Strand cDNA Synthesis Kit (Qiagen Ltd. Crawley, UK) using oligo dT primers following the manufactures instructions. After the cDNA synthesis, the final volume was adjusted to 120 µL with nuclease free water.

#### Quantitative Real-Time PCR

qPCR was performed on a select panel of cytokines including; *IL1A*, *IL1B*, *IL4*, *IL6*, *IL8*, *IL10*, *IL17*, *IL21*, Interferon (*INFG*), Tumor Necrosis Factor (*TNF*), transforming growth factor (*TGFB*), Forkhead box P3 (*FOXP3*), nutrient transporters including, Fatty acid binding protein (*FABP*), Glucose transporter 2 (*GLUT2*), Peptide transporter 1 (*PEPT1*) and tight junction protein Occludin (*OCLN*). All primers were designed using Primer Express™ software and were synthesised by MWG Biotech (Milton Keynes, UK) and are presented in Table 3, all assays had efficiencies in the range of 90–110%. Dissociation analysis confirmed the specificity of the resulting PCR products. Glyceraldehyde 3-phosphate dehydrogenase (*GAPDH*), β_2_ microglobulin (*B2M*), Beta-actin (*ACTB*), Peptidylprolyl isomerase A (*PPIA*) and 14-3-3 protein zeta/delta (*YWHAZ*) were used as endogenous controls. Quantitative PCR was carried out using 96 well fast optical plates on a 7500HT ABI Prism Sequence Detection System (Applied Biosystems, Foster City, CA) using Fast SYBR Green PCR Master Mix (Applied Biosystems). All reactions were performed in triplicate in a total volume of 20 µL containing 10 µL Fast SyBr PCR Master mix, 1 µL forward and reverse primer (5 µM) and 8 µL water and 1 µL of template cDNA. The thermal cycling conditions were as follows, 95 °C for 10 min, 40 cycles of 95 °C for 15 s and 65 °C for 1 min.

**Table 3:** Oligonucleotide sequence of forward and reverse primers used in all qPCR assays.

Gene	Accession number	Forward primer (5′-3′)	Tm(°C)	Product Length (bp)
		Reverse primer (5′-3′)		
***Reference genes***				
*ACTB*	XM_001928093.1	GCACGGCATCATCACCAA	52.75	70
		CCGGAGCTCGTTGTAGAAGGT	55.99	
*PPIA*	NM_214353.1	CGGGTCCTGGCATCTTGT	62.1	75
		TGGCAGTGCAAATGAAAAACT	60.7	
*GAPDH*	AF017079.1	CAGCAATGCCTCCTGTACCA	62.2	72
		ACGATGCCGAAGTTGTCATG	62.1	
***Target genes***				
*IL1A*	NM_214029.1	CAGCCAACGGGAAGATTCTG	63.0	76
		ATGGCTTCCAGGTCGTCAT	60.49	
*IL1B*	NM_001005149.1	TTGAATTCGAGTCTGCCCTGT	60.59	76
		CCCAGGAAGACGGGCTTT	60.94	
*IL4*	HQ236500.1	CCAACCCTGGTCTGCTTACTG	61.8	71
		TTGTAAGGTGATGTCGCACTTGT	58.9	
*IL6*	AB194100	AGACAAAGCCACCACCCCTAA	55.27	69
		CTCGTTCTGTGACTGCAGCTTATC	59.92	
*IL8*	NM_213867.1	TGCACTTACTCTTGCCAGAACTG	61.9	82
		CAAACTGGCTGTTGCCTTCTT	61.7	
*IL10*	NM_214041.1	GCCTTCGGCCCAGTGAA	63.4	71
		AGAGACCCGGTCAGCAACAA	63.1	
*IL17*	NM_001005729.1	CCCTGTCACTGCTGCTTCTG	60.57	57
		TCATGATTCCCGCCTTCAC	60.40	
*IL21*	NM_214415	GGCACAGTGGCCCATAAATC	57.38	124
		GCAGCAATTCAGGGTCCAAG	61.51	
*INFG*	NM_213948.1	TCTAACCTAAGAAAGCGGAAGAGAA	61.12	81
		TTGCAGGCAGGATGACAATTA	61.54	
*FOXP3*	NM_001128438.1	GTGGTGCAGTCTCTGGAACAAC	60.57	68
		AGGTGGGCCTGCATAGCA	61.18	
*TNF*	NM_214022.1	TGGCCCCTTGAGCATCA	62.5	68
		CGGGCTTATCTGAGGTTTGAGA	62.8	
*TGFB*	NM_214015.1	AGGGCTACCATGCCAATTTCT	60.63	101
		CGGGTTGTGCTGGTTGTACA	61.68	
*FABP*	NM_001031780.1	TCGGGATGAAATGGTCCAGACT	62.4	102
		TGTGTTCTGGGCTGTGCTCCA	61.8	
*PEPT1*	NM_214347.1	GGATAGCCTGTACCCCAAGCT	61.8	73
		CATCCTCCACGTGCTTCTTGA	59.8	
*GLUT2*	AF054835.1	CCAGGCCCCATCCCCTGGTT	65.5	96
		GCGGGTCCAGTTGCTGAATGC	63.7	
*OCLN*	F1SK31	CGGTGAGAAGATTGGCTGAT	62.3	100
		TTTCAAAAGGCCTGGATGAC	62.7	

#### Normalization of data

Mean C_t_ values were converted to relative quantities using the formula, Relative quantity = 2^−ΔCt^, where ΔC_t_ is the change in the C_t_ values of the sample relative to the highest expression (minimum C_t_ value). Relative quantities for the endogenous controls were imported into geNorm^27^ and a normalization factor was derived. *B2M* and *ACTB* had the lowest M value of all the reference genes tested and were therefore chosen for subsequent normalization. The relative quantities for the target genes were then divided by the normalization factor to give the final normalized relative quantity for each target gene in each sample.

### Laboratory analysis of feed

The feed samples were milled through a 1mm screen (Christy and Norris hammer mill, Ipswich, UK). The dry matter (DM) of the feed was determined after drying at 103 °C for a minimum of 16 h. Ash was determined after ignition of a known weight of concentrate in a muffle furnace (Nabertherm, Bremen, Germany) at 500 °C for 4 h. The crude protein (CP) content was determined as Kjeldahl N × 6.25 using the LECO FP 528 instrument. The neutral detergent fibre (NDF) content was determined according to Van Soest *et al*.^28^.

### Statistical analysis

The data were initially checked for normality using the UNIVARIATE procedure of SAS. The growth performance and faecal scores were analysed by repeated measures analysis using PROC MIXED procedure of SAS^29^. The model included the fixed effects of yeast β-glucan, 5kDaR, time and the associated two and three way interactions while the random effect was pen. Initial body weight was used as a covariate for growth performance data. The data on microbial population and gene expression were analysed using PROC MIXED procedure of SAS. The model included yeast β-glucan, 5kDaR and the associated two way interaction and the random effect was pen. Contrast statements were used to compare (1) T1 and T3 vs. T2 and T4 – non-5kDaR-supplemented vs. 5kDaR-supplemented pigs (5kDaR effect), (2) T1 and T2 vs. T3 and T4 – non-yeast-supplemented vs. yeast-supplemented pigs (yeast β-glucan effect), (3) the interaction between the 5kDaR effect and yeast β-glucan effect. Additionally contrast statements were used to compare ZnO (T5) with the control diet (T1) (Contrast 4) and with the combination of yeast β-glucan + 5kDaR inclusion (T4) (Contrast 5). The individual pen served as the experimental unit for all variables measured. Least square means were computed, and P*-*values were adjusted for multiple comparisons using the Tukey-Kramer adjustment. The mean values were considered to be significantly different when P < 0.05 and considered a numerical tendency when P < 0.10. Least square means are reported with pooled standard errors.

## Results

### Faecal score

**Figure 1:**
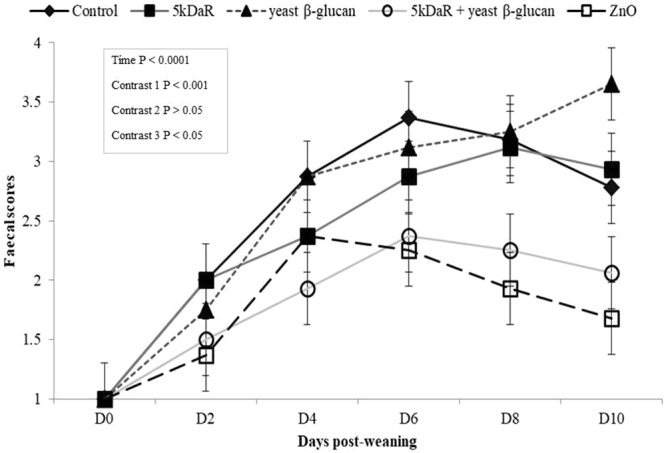
Effect of dietary treatments on faecal scores from 0 d up to 10 d post-weaning. Values are LS means, with their standard errors represented by vertical bars. Scale from 1 to 5: (1) hard, firm faeces; (2) slightly soft faeces; (3) soft, partially formed faeces; (4) loose, semi-liquid faeces; and (5) watery, mucous-like faeces (Pierce *et al*., 2007). The dietary treatments are represented as: (**-♦-**) control diet, (-■-) 5kDaR diet, (--▲--) yeast β-glucan diet, (-**Ο**-) 5kDaR + yeast β-glucan diet and (-□-) ZnO diet. The contrast statement used and statistical significance values achieved are as follows: Contrast 1, the effect of 5kDaR vs. non 5kDaR diet (P < 0.001); Contrast 2, the effect of yeast β-glucan vs. non yeast β-glucan diet (P > 0.05); Contrast 3: the interaction between 5kDaR and yeast β-glucan (P < 0.05); Contrast 4, the effect of ZnO vs control diet (P < 0.01); and Contrast 5: the ZnO vs. 5kDaR and yeast β-glucan (P > 0.05).

The effects of dietary treatments on faecal scores are presented in Fig. 1. A healthy gut is associated with faecal scores between 2.0 to 2.5 whereas faecal scores of 3.0 to 4.0 represent diarrhoea scores and 4.5 to 5.0 represent severe diarrhoea that requires antibiotic intervention. While there was no yeast β-glucan x 5kDaR x time interaction on faecal scores (P > 0.05); a time effect on faecal scores was evident. There was an interaction between yeast β-glucan x 5kDaR on faecal scores (P < 0.05; Contrast 3); the inclusion of 5kDaR alone or yeast β-glucan alone had no effect on faecal scores compared to the control diet, while the combination of 5kDaR + yeast β-glucan decreased faecal scores compared to the individual supplements. The inclusion of ZnO decreased faecal scores relative to the control treatment (P < 0.05; Contrast 4) while faecal scores were similar between ZnO and the combination of 5kDaR + yeast β-glucan (P > 0.05; Contrast 5) throughout the experimental period.

### Growth parameters

The effects of dietary supplements on body weight (at d 10), average daily gain (ADG), average daily feed intake (ADFI) and gain to feed ratio (G:F) are presented in Table 4. There was no yeast β-glucan x 5kDaR x time interaction on body weight, ADG, ADFI or G:F. There was a significant time effect on ADG (P < 0.05) and ADFI (P < 0.001). There was an interaction between yeast β-glucan x 5kDaR on body weight and ADG (P < 0.05; Contrast 3); the inclusion of 5kDaR alone or yeast β-glucan alone had no effect on body weight or ADG compared to the control diet, while the combination of 5kDaR + yeast β-glucan increased bodyweight and ADG compared to the individual supplements. The inclusion of ZnO increased body weight (P < 0.05), ADG (P < 0.01) and ADFI (P < 0.05) compared to the control group (Contrast 4) while these parameters were similar between the piglets offered ZnO and the combination of 5kDaR + yeast β-glucan (P > 0.05; Contrast 5).

**Table 4:** Effect of feed supplements on body weight, average daily gain (ADG), average daily feed intake (ADFI) and gain to feed ratio (G:F) in weaning pigs (LSM ± SEM).

5kDaR	No	Yes	No	Yes	No	SEM	Significance				
yeast β-glucan	No	No	Yes	Yes	No						
ZnO	—	—	—	—	Yes		Contrast 1^a^	Contrast 2^b^	Contrast 3^c^	Contrast 4^d^	Contrast 5^e^
Body weight D 10 (kg)	7.58	7.56	7.4	8.25	8.27	0.227	*0*.*0552*	*0*.*111*	*0*.*0500*	*0*.*0233*	*0*.*9431*
ADG 0–10 (kg/day)	0.022	0.030	−0.003	0.112	0.127	0.0306	*0*.*0452*	*0*.*3466*	*0*.*0499*	*0*.*0058*	*0*.*7343*
ADFI 0–10 (kg/day)	0.276	0.338	0.342	0.360	0.369	0.0247	*0*.*1459*	*0*.*1036*	*0*.*4158*	*0*.*0148*	*0*.*7959*
G:F 0–10 (kg/kg)	0.081	0.087	0.136	0.247	0.272	0.119	*0*.*561*	*0*.*493*	*0*.*6316*	*0*.*1808*	*0*.*2919*

^a^Contrast 1: the effect of 5kDaR vs. non 5kDaR diet.

^b^Contrast 2: the effect of yeast β-glucan vs. non yeast β-glucan diet.

^c^Contrast 3: the interaction between 5kDaR and yeast β-glucan.

^d^Contrast 4: the effect of ZnO vs. control diet.

^e^Contrast 5: the ZnO vs 5kDaR and yeast β-glucan.

### Microbiology

The effects of dietary supplements on the abundance of a selected panel of bacterial groups in the caecal and colonic digesta are presented in Table 5.

**Table 5:** Effect of feed additives on log transformed gene copy number/g dry matter of digesta of a panel of selected bacterial groups present in caecal and colonic digesta (LSM ± SEM).

5kDaR	No	Yes	No	Yes	No	SEM	Significance				
yeast β-glucan	No	No	Yes	Yes	No						
ZnO	—	—	—	—	Yes		Contrast 1^a^	Contrast 2^b^	Contrast 3^c^	Contrast 4^d^	Contrast 5^e^
***Caecal digesta***											
Total bacteria	12.40	12.83	12.46	12.69	12.65	0.1804	*0*.*0905*	*0*.*8499*	*0*.*5887*	*0*.*2922*	*0*.*8489*
*Bacteroidetes*	11.71	12.56	11.49	12.42	12.60	0.3295	*0*.*0281*	*0*.*6364*	*0*.*9168*	*0*.*0500*	*0*.*6679*
*Firmicutes*	12.15	12.27	12.12	12.27	12.16	0.0975	*0*.*1483*	*0*.*8626*	*0*.*8611*	*0*.*9210*	*0*.*3544*
*Bifidobacterium spp*.	9.37	9.09	8.99	9.22	8.94	0.1147	*0*.*8491*	*0*.*3283*	*0*.*0500*	*0*.*0080*	*0*.*0500*
*Enterobacteriaceae*	10.62	11.11	11.69	11.07	9.96	0.4822	*0*.*8964*	*0*.*3143*	*0*.*2834*	*0*.*3135*	*0*.*0713*
*Lactobacillus spp*.	12.41	12.12	11.99	12.30	12.31	0.2382	*0*.*9489*	*0*.*6094*	*0*.*2161*	*0*.*7673*	*0*.*9661*
AEEC strain	9.28	9.52	10.49	9.36	8.99	0.2030	*0*.*0401*	*0*.*0172*	*0*.*0029*	*0*.*2957*	*0*.*1506*
***Colonic digesta***											
Total bacteria	13.23	13.33	13.33	13.34	13.37	0.1094	*0*.*5936*	*0*.*6276*	*0*.*6943*	*0*.*3365*	*0*.*8237*
*Bacteroidetes*	11.96	11.88	12.15	11.36	12.26	0.4039	*0*.*3153*	*0*.*7032*	*0*.*4073*	*0*.*5923*	*0*.*0898*
*Firmicutes*	11.97	12.11	12.08	12.09	12.13	0.1085	*0*.*4891*	*0*.*7032*	*0*.*5982*	*0*.*2829*	*0*.*7676*
*Bifidobacterium spp*.	9.45	9.15	8.87	8.95	8.96	0.1856	*0*.*5562*	*0*.*0407*	*0*.*2991*	*0*.*0500*	*0*.*9598*
*Enterobacteriaceae*	10.37	10.73	11.37	10.57	9.64	0.4222	*0*.*6270*	*0*.*6317*	*0*.*2136*	*0*.*2542*	*0*.*1214*
*Lactobacillus spp*.	10.82	10.41	10.80	10.52	11.12	0.2499	*0*.*1946*	*0*.*8639*	*0*.*8095*	*0*.*4201*	*0*.*0886*
AEEC strain	9.22	9.23	9.86	9.13	9.08	0.2536	*0*.*1792*	*0*.*3033*	*0*.*1631*	*0*.*7066*	*0*.*8893*

^a^Contrast 1: the effect of 5kDaR vs. non 5kDaR diet.

^b^Contrast 2: the effect of yeast β-glucan vs. non yeast β-glucan diet.

^c^Contrast 3: the interaction between 5kDaR and yeast β-glucan.

^d^Contrast 4: the effect of ZnO vs. control diet.

^e^Contrast 5: the ZnO vs 5kDaR and yeast β-glucan.

#### Caecal digesta

There was a yeast β-glucan x 5kDaR interaction observed on the abundance of *Bifidobacterium spp*. and Attaching and Effacing *Escherichia coli* (AEEC) strains (P < 0.05; Contrast 3). The inclusion of yeast β-glucan increased the abundance of AEEC strains and decreased the abundance of *Bifidobacterium spp*. compared to the control diet, however the abundance of AEEC strains and *Bifidobacterium spp*. were not altered when yeast was added to the 5kDaR diet. The piglets offered diets supplemented with 5kDaR had increased *Bacteroidetes* (12.498 vs. 11.607 ± 0.255, P < 0.05) compared to the diets without 5kDaR inclusion (Contrast 1). The inclusion of ZnO in the diet increased the *Bacteroidetes* abundance and decreased the abundance of *Bifidobacterium spp*. (P < 0.05) compared to the control (Contrast 4) and the combination of 5kDaR + yeast β-glucan diets (P < 0.05; Contrast 5).

#### Colonic digesta

There was no interaction between yeast β-glucan x 5kDaR on the selected microbial populations (Contrast 3). Similar to the caecum, dietary inclusion of yeast β-glucan decreased the abundance of *Bifidobacterium spp*. compared to non yeast β-glucan diets (8.911 vs. 9.302 ± 0.127, P < 0.05). The inclusion of ZnO in the diet decreased the abundance of *Bifidobacterium spp*. compared to the control diet (P < 0.05).

### Gene expression analysis of duodenum, ileum and colon

The effect of dietary supplements on the normalized relative expression of a selected panel of genes expressing cytokines (*IL1A*, *IL1B*, *IL4*, *IL6*, *IL8*, *1L10*, *IL17*. *INFG*, *TNF*, *TGFB*), nutrient transporters (*FABP*, *PEPT1*, *GLUT2*) and tight junction proteins (*OCLN*) in the duodenum and ileum are presented in Table 6.

**Table 6:** Effect of dietary treatments on relative quantity (RQ) of a panel of selected cytokine and nutrient transporter gene expression in porcine colonic tissues (LSM ± SEM).

5kDaR	No	Yes	No	Yes	—	SEM	Significance				
yeast β-glucan	No	No	Yes	Yes	—						
ZnO	—	—	—	—	Yes		Contrast 1^a^	Contrast 2^b^	Contrast 3^c^	Contrast 4^d^	Contrast 5^e^
***Duodenum***											
*IL1A*	1.224	0.764	1.290	0.890	0.900	0.1300	*0*.*0033*	*0*.*4430*	*0*.*8087*	*0*.*0953*	*0*.*9594*
*IL1B*	1.400	0.948	1.227	0.930	1.075	0.1761	*0*.*0602*	*0*.*6089*	*0*.*6789*	*0*.*3293*	*0*.*6419*
*IL8*	1.164	0.706	1.217	0.948	0.778	0.0019	*0*.*0050*	*0*.*2079*	*0*.*4154*	*0*.*0216*	*0*.*2854*
*IL10*	1.165	1.148	0.948	1.122	0.828	0.1626	*0*.*6502*	*0*.*4875*	*0*.*5844*	*0*.*1825*	*0*.*2416*
*IL17*	1.224	1.046	1.232	1.040	0.876	0.1048	*0*.*1478*	*0*.*9950*	*0*.*9523*	*0*.*0228*	*0*.*2815*
*INFG*	1.308	0.785	1.066	0.920	0.792	0.1571	*0*.*0747*	*0*.*7647*	*0*.*3002*	*0*.*0443*	*0*.*6442*
*TNF*	1.130	1.063	0.951	0.878	1.082	0.1376	*0*.*5860*	*0*.*1638*	*0*.*9776*	*0*.*8199*	*0*.*3317*
*TGFB*	1.241	1.175	1.060	1.022	0.744	0.0905	*0*.*5944*	*0*.*0990*	*0*.*8837*	*0*.*0004*	*0*.*0421*
*PEPT1*	0.950	1.097	1.024	1.234	1.152	0.1200	*0*.*1592*	*0*.*3997*	*0*.*8018*	*0*.*2413*	*0*.*6349*
*GLUT2*	1.018	1.138	1.017	1.040	1.000	0.1210	*0*.*5805*	*0*.*6981*	*0*.*7063*	*0*.*9213*	*0*.*8316*
***Ileum***											
*IL1A*	1.080	0.855	0.946	0.954	0.678	0.1168	*0*.*3174*	*0*.*7631*	*0*.*2899*	*0*.*0229*	*0*.*1248*
*IL1B*	0.996	0.808	1.316	0.988	0.454	0.1735	*0*.*1611*	*0*.*2957*	*0*.*7728*	*0*.*0466*	*0*.*498*
*IL8*	1.078	0.861	1.140	0.778	0.662	0.1183	*0*.*1336*	*0*.*6127*	*0*.*7779*	*0*.*0292*	*0*.*5222*
*IL10*	1.313	1.136	0.991	0.888	0.804	0.1253	*0*.*2677*	*0*.*0219*	*0*.*5705*	*0*.*0112*	*0*.*6423*
*IL17*	0.915	0.928	0.806	0.795	0.740	0.1054	*0*.*8235*	*0*.*4384*	*0*.*4100*	*0*.*2121*	*0*.*7214*
*INFG*	1.310	0.716	0.968	0.798	0.838	0.2488	*0*.*1388*	*0*.*6056*	*0*.*4009*	*0*.*2138*	*0*.*9130*
*TNF*	1.088	1.148	0.767	1.067	0.810	0.1056	*0*.*3111*	*0*.*2448*	*0*.*4808*	*0*.*0835*	*0*.*0985*
*TGFB*	1.158	1.366	1.024	1.041	1.045	0.1549	*0*.*7151*	*0*.*3262*	*0*.*2002*	*0*.*6478*	*0*.*9893*
*PEPT1*	0.905	0.960	1.013	0.928	1.580	0.2420	*0*.*8196*	*0*.*8355*	*0*.*8342*	*0*.*0424*	*0*.*0577*
*GLUT2*	0.610	0.853	0.730	1.234	1.093	0.1439	*0*.*0493*	*0*.*0930*	*0*.*6340*	*0*.*0366*	*0*.*5114*
***Colon***											
*IL1A*	1.951	1.395	1.776	1.002	1.021	0.4172	*0*.*1440*	*0*.*5246*	*0*.*8068*	*0*.*1271*	*0*.*9739*
*IL1B*	1.779	1.393	1.996	1.285	1.216	0.3862	*0*.*1388*	*0*.*5246*	*0*.*6056*	*0*.*2120*	*0*.*9739*
*IL8*	1.683	0.652	2.913	1.252	0.930	0.3890	*0*.*0041*	*0*.*0393*	*0*.*4574*	*0*.*2041*	*0*.*5683*
*IL10*	1.540	1.156	0.860	0.961	0.956	0.3124	*0*.*6726*	*0*.*2000*	*0*.*4695*	*0*.*2109*	*0*.*9909*
*IL17*	1.731	1.513	0.830	0.925	0.923	0.3516	*0*.*8693*	*0*.*5770*	*0*.*6765*	*0*.*1166*	*0*.*9974*
*INFG*	2.238	1.090	1.200	0.966	0.923	0.2672	*0*.*0013*	*0*.*0035*	*0*.*5200*	*0*.*0018*	*0*.*1753*
*TNF*	1.498	0.894	1.493	0.752	0.838	0.3062	*0*.*0649*	*0*.*8340*	*0*.*8444*	*0*.*1593*	*0*.*8394*
*TGFB*	1.883	1.441	0.938	1.034	1.328	0.2947	*0*.*5669*	*0*.*0334*	*0*.*3759*	*0*.*2157*	*0*.*4936*

The targets *IL4*, *IL6*, *IL21*, *FABP*, *FOXP3* and *OCLN* expression were undetectable in all the analysed tissue samples. *PEPT1* and *GLUT2* expression were undetectable in colonic tissues.

^a^Contrast 1: the effect of 5kDaR vs. non 5kDaR diets.

^b^Contrast 2: the effect of yeast β-glucan vs. non yeast β-glucan diets.

^c^Contrast 3: the interaction between 5kDaR and yeast β-glucan.

^d^Contrast 4: the effect of ZnO vs. control diet.

^e^Contrast 5: the ZnO vs 5kDaR and yeast β-glucan.

#### Duodenum

There was no interaction between yeast β-glucan x 5kDaR on the selected panel of genes examined in the duodenal tissue (Contrast 3). Inclusion of 5kDaR in the diet suppressed the expression of *IL1A* (0.827 vs. 1.257 ± 0.085, P < 0.01), *IL8* (0.827 vs. 1.190 ± 0.080, P < 0.05) and a tendency to suppress *IL1B* (0.939 vs. 1.313 ± 0.121, P < 0.06) and *INFG* (0.993 vs. 1.046 ± 0.121, P < 0.07) compared to the diets without 5kDaR supplementation (Contrast 1). The inclusion of yeast β-glucan had no effect (P > 0.05) on the selected panel of genes examined in the duodenum.

The inclusion of ZnO in diet suppressed the expression of *IL8*, *IL17*, *INFG* and *TGFB* (P < 0.05) compared to control diet (Contrast 4). The expression levels of the selected panel of genes were similar between the ZnO diet and yeast β-glucan + 5kDaR diet, except for *TGFB*, which was decreased in the ZnO diet (P < 0.05).

#### Ileum

There were no interactions observed between yeast β-glucan x 5kDaR on the selected panel of genes (Contrast 3). The inclusion of 5kDaR in the diet increased the expression of *GLUT2* compared to diets without 5kDaR supplementation (1.012 vs. 0.707 ± 0.101, P < 0.05). The yeast β-glucan inclusion suppressed the expression of *IL10* (0.933 vs. 1.242 ± 0.0899, P < 0.05) compared to diets without yeast β-glucan supplementation. Zinc oxide suppressed the expression of *IL1A*, *IL1B*, *IL8* and *IL10* and increased the expression of *PEPT1* and *GLUT2* (P < 0.05) compared to the control diet. The expression levels of the selected panel of genes were similar between the ZnO diet and yeast β-glucan + 5kDaR diet, other than a numerical increase in PEPT1 (P < 0.06) in the ZnO diet.

#### Colon

There was no yeast β-glucan x 5kDaR interactions observed on the selected panel of genes in the colonic tissues. The inclusion of 5kDaR in diet suppressed the expression of *IL8* (0.952 vs. 2.298 ± 0.2914; P < 0.01) and *INFG* (0.743 vs. 1.719 ± 0.1852; P < 0.01) and numerically decreased *TNF* (0.823 vs. 1.495 ± 0.240; P < 0.06) compared to non-5kDaR diets. The inclusion of yeast β-glucan increased the expression of *IL8* (2.083 vs. 1.167; P < 0.01) and decreased the expression of *INFG* (0.798 vs. 1.664 ± 0.1830; P < 0.05) and *TGFB* (0.986 vs. 1.662 ± 0.2130; P < 0.01) compared to non-yeast β-glucan diets. The inclusion of ZnO suppressed the expression of *INFG* (P < 0.01) compared to the control diet. The expression levels of the selected panel of genes were similar between the ZnO diet and yeast β-glucan + 5kDaR diet.

## Discussion

This study examined the potential health benefits of dietary inclusion of a sodium caseinate derived milk hydrolysate (5kDaR) and a yeast β-glucan, individually or in combination, in piglets following early and abrupt separation from the sow. These dietary supplements were compared to the recommended pharmacological dose of zinc oxide (ZnO) and evaluated on parameters including faecal scores, growth performance and the re-establishment of gut homeostasis (microbial composition and mucosal inflammation). While numerous studies have explored the potential benefits of different feed supplements following abrupt weaning, no single compound has been able to replace ZnO. There is, however, imminent pressure to eliminate the use of heavy metals in pig diets^13^. In the current study, the individual inclusion of either 5kDaR or yeast β-glucan did not support the re-establishment of gut homeostasis during the post-weaning challenge period. However, supplementing the piglet diet with a combination of yeast β-glucan + 5kDaR suppressed post-weaning diarrhoea, improved faecal scores, growth parameters and suppressed the expression of inflammatory gene markers in duodenal and ileal tissues – effects comparable to those observed in piglets supplemented with the pharmacological dose of ZnO.

Post-weaning diarrhoea is a consequence of decreased digestive and absorptive capacity of the small intestine that leads to an increase in pathogenic bacterial numbers in the large intestine^30^. An established method of evaluating gut health of a newly weaned piglet is by scoring their faeces based on consistency^25^. This is also accepted as a criterion for claims on maintenance of normal gut function for humans by the European Food Safety Authority^31^. In this study, piglets supplemented with pharmacological doses of ZnO maintained healthy faecal scores of approximately 2.0 to 2.5 throughout the experimental period. In contrast, the pigs receiving the basal diet or individual inclusion of 5kDaR or yeast β-glucan showed signs of diarrhoea (faecal score range 3.0 to 4.0) from day 2–3 until day 9–10 post-weaning. In contrast, the combination of yeast β-glucan + 5kDaR restored healthy functioning of the gut during the post-weaning period similar to ZnO supplementation.

In the current study, piglets receiving the basal diet had higher numbers of *Enterobacteriaceae* and AEEC strains in caecal and colonic digesta along with diarrhoea/raised faecal scores. A plausible explanation for this might be the high crude protein concentration in the diet, a high soybean meal inclusion level and a low level of lactose^32^, which can disrupt gut homeostasis in the weaned piglet. In a previous study, the high level of dietary crude protein predisposed the piglet to post-weaning colibacillosis^33^. In contrast, supplementation of the basal diet with the combination of yeast β-glucan + 5kDaR maintained healthy faecal scores with lower abundance of pathogenic bacteria in the digesta.

The maintenance of a healthy gut, by supplementation with yeast β-glucan + 5kDaR combination and ZnO, was not only evident through improved faecal scores, but these piglets also had improved growth parameters (ADG, ADFI and body weights on d 10). In contrast, the individual inclusion of 5kDaR or yeast β-glucan had no effect on the measured growth parameters. Dong & Pluske^34^ clearly identified the necessity to improve feed intake immediately post-weaning, as low feed intake, which is synonymous with commercial weaning practices, leads to disrupted gut morphology, poor growth rate and higher risk of disease. In the current study, both yeast β-glucan + 5kDaR and ZnO supplemented pigs had higher ADFI than the pigs fed the basal diet. While commercial weaning negatively impacts the absorptive capacity of the small intestine^30^; the ZnO and yeast β-glucan + 5kDaR supplemented piglets had increased expression of *GLUT2* and *PEPT1* in the ileum. Glucose transporter 2 is widely known to facilitate the absorption of glucose in the small intestine^35^, and was recently associated with maintenance of gut homeostasis in rats^36^. The intestinal absorption of hydrolysed proteins is facilitated by *PEPT1*; in fact *PEPT1* also has a role in gut homeostasis^37^. The increased expression of *GLUT2* and *PEPT1* is most likely related to a higher availability of glucose and amino acids in the small intestine as well as increased villus surface area, as a consequence of the higher ADFI in both the yeast β-glucan + 5kDaR and ZnO groups.

The gut microbiota plays an important role in maintaining gut homeostasis in both humans and animals^38,39^. Four important phyla in the caecum and colon of healthy pigs are *Bacteroidetes*, *Firmicutes*, *Actinobacteria and Proteobacteria*^40–42^. The main role of *Bacteroidetes* and *Firmicutes* is to ferment the undigested fibre in the distal part of the gastrointestinal tract. The phylum *Bacteroidetes* is further associated with gastrointestinal tract development, activation of T cell mediated immune response and limiting the colonization of the gut with pathogenic bacteria^43^. In this study, the inclusion of both ZnO and 5kDaR was associated with an increase in *Bacteroidetes* in the caecal digesta. An important member of the *Actinobacteria* phylum is the *Bifidobacterium spp*., because of its probiotic potential^44^. This species was decreased with the inclusion of ZnO and yeast β-glucan. Interestingly, the yeast β-glucan + 5kDaR combination did not negatively influence the abundance of the analyzed gut bacteria and was associated with higher abundance of the *Bifidobacterium spp*. A more indepth metagenomic analysis of the gut microbiome would be beneficial to confirm that the combination diet does indeed have an enhanced beneficial microbial effect over ZnO.

Within the *Proteobacteria* phylum, the abundance of *Enterobacteriaceae* family and AEEC strains were measured in this study. The inclusion of yeast β-glucan in the newly weaned piglet was associated with a significant increase in the pathogenic AEEC strains in the large intestine, which may explain the diarrhoeal faecal scores, the lower performance and increased inflammation of the gut in the piglets. This appears contradictory to a previous study that identified that the abundance of *Enterobacteriaceae* was decreased following dietary inclusion of similar yeast β-glucan in mature healthy 19 kg pigs^23^. This variation in response to β-glucans may reflect the difference in the status of the piglet immune system at these different stages of development. Abrupt commercial weaning normally occurs during a time period where the piglet is partially dependent on maternal imunoglobulins, via the milk, and does not yet have a fully developed adaptive immune system. In contrast to a more mature pig, yeast β-glucan may not have a beneficial effect during this time-period as it elicits its response against pathogens by activating immune cells such as granulocytes, monocytes and macrophages^45^. This suggests that consideration must be given to the stage of development of the pig while exploring the options of dietary supplementation.

Homeostasis of the gut is dependent on the orchestration of a signaling network that exists between the intestinal epithelial cells, gut microbiota and the local immune cells. Disruption of this regulatory mechanism can lead to chronic intestinal inflammation, as observed in pigs^12^ and humans^43^. The plausible role of ZnO in maintaining gut homeostasis during abrupt weaning in the pig, by suppressing the inflammatory nuclear factor κB (NFκB) pathway^46^, is clear in this experiment. The inclusion of both ZnO and the combination of yeast β-glucan + 5kDaR was associated with a decrease in the pro-inflammatory cytokines *IL1A*, *IL1B*, *IL8*, *IL17* and *INFG* expression in both duodenal and ileal tissues. Similar effects of ZnO on the NFκB pathway were shown previously by Bouwhuis *et al*.^47^. This suggests that the combination of yeast β-glucan + 5kDaR also suppresses of the NFκB pathway. The inclusion of 5kDaR suppressed the expression of pro-inflammatory *IL1A* and *IL8* genes in the duodenal tissue; however this suppression was no longer evident in the ileum and only evident for *IL8* in the colon, indicating a gradual breakdown of the bioactive molecules during transit through the small intestine. In contrast, the inclusion of yeast β-glucan alone in the diet had no effect on the immune gene expression profile in the duodenal or ileal tissues, with increased *IL8* expression in the colon.

An interesting finding from this study was the synergistic interaction observed between the yeast β-glucan and the 5kDaR. While it was not within the scope of this study to explore the biochemical nature of this interaction, one possibility is that the β-glucan acted as a microencapsulating agent for the milk hydrolysate, thus preserving its bioactivity *in-vivo*. Beta-glucans, from a range of sources including yeast^48,49^, oats^50^ and barley^22^ have properties that make them suitable encapsulation agents. More in-depth studies are warranted to identify how the current combination of supplements exerts this protective function in the gastric environment.

## Conclusions

The combination of 5kDaR + yeast β-glucan supplementation in the weaning pig diet was associated with an improvement in overall performance and gut health parameters (faecal consistency, microbiota and inflammation) that are, at least, comparable to ZnO. This study also suggests that yeast β-glucan may have a protective function for proteins in the gastric environment worthy of further exploration.

## References

[1] Lallès JP, Bosi P, Smidt H, Stokes CR (2007). Weaning—a challenge to gut physiologists. Livestock Science.

[2] Boudry G, Péron V, Le Huërou-Luron I, Lallès JP, Sève B (2004). Weaning induces both transient and long-lasting modifications of absorptive, secretory, and barrier properties of piglet intestine. The Journal of nutrition.

[3] Inoue R, Tsukahara T, Nakanishi N, Ushida K (2005). Development of the intestinal microbiota in the piglet. The Journal of general and applied microbiology.

[4] Madec F (2000). Experimental models of porcine post-weaning colibacillosis and their relationship to post-weaning diarrhoea and digestive disorders as encountered in the field. Veterinary microbiology.

[5] Castillo M, Martín-Orúe SM, Nofrarías M, Manzanilla EG, Gasa J (2007). Changes in caecal microbiota and mucosal morphology of weaned pigs. Veterinary microbiology.

[6] Sargeant HR, McDowall KJ, Miller HM, Shaw MA (2010). Dietary zinc oxide affects the expression of genes associated with inflammation: transcriptome analysis in piglets challenged with ETEC K88. Veterinary immunology and immunopathology.

[7] Pérez VG (2011). Additivity of effects from dietary copper and zinc on growth performance and fecal microbiota of pigs after weaning 1 2. Journal of animal science.

[8] McAlpine P, O’Shea CJ, Varley PF, Flynn B, O’Doherty JV (2012). The effect of seaweed extract as an alternative to zinc oxide diets on growth performance, nutrient digestibility, and fecal score of weaned piglets. Journal of animal science,.

[9] Sales J (2013). Effects of pharmacological concentrations of dietary zinc oxide on growth of post-weaning pigs: a meta-analysis. Biological trace element research.

[10] Heim G (2014). Effect of seaweed-derived laminarin and fucoidan and zinc oxide on gut morphology, nutrient transporters, nutrient digestibility, growth performance and selected microbial populations in weaned pigs. British Journal of Nutrition.

[11] Lalles JP, Bosi P, Smidt H, Stokes CR (2007). Nutritional management of gut health in pigs around weaning. Proceedings of the Nutrition Society.

[12] Sweeney T, O’Doherty JV (2016). Marine macroalgal extracts to maintain gut homeostasis in the weaning piglet. Domestic animal endocrinology.

[13] O’Doherty JV, Bouwhuis MA, Sweeney T (2017). Novel marine polysaccharides and maternal nutrition to stimulate gut health and performance in post-weaned pigs. Animal Production Science.

[14] Mukhopadhya, A., & Sweeney, T. Milk Proteins: Processing of Bioactive Fractions and Effects on Gut Health. In Milk Proteins-From Structure to Biological Properties and Health Aspects. InTech (2016).

[15] Samuelsen ABC, Schrezenmeir J, Knutsen SH (2014). Effects of orally administered yeast‐derived beta‐glucans: A review. Molecular nutrition & food research.

[16] Zhu F, Du B, Xu B (2016). A critical review on production and industrial applications of beta-glucans. Food Hydrocolloids.

[17] Ting Y, Jiang Y, Ho CT, Huang Q (2014). Common delivery systems for enhancing *in vivo* bioavailability and biological efficacy of nutraceuticals. Journal of Functional Foods.

[18] Mukhopadhya A (2015). The anti-inflammatory potential of a moderately hydrolysed casein and its 5 kDa fraction in *in vitro* and *ex vivo* models of the gastrointestinal tract. Food & function.

[19] Mukhopadhya A, Sweeney T, O’Shea C, O’Doherty JV (2016). A comparative study of alternatives to pharmacological doses of zinc for improving gut health parameters in weaning piglets. Journal of Animal Science,.

[20] Singh MN, Hemant KSY, Ram M, Shivakumar HG (2010). Microencapsulation: A promising technique for controlled drug delivery. Research in pharmaceutical sciences.

[21] De Smet R, Allais L, Cuvelier CA (2014). Recent advances in oral vaccine development: Yeast-derived β-glucan particles. Human vaccines & immunotherapeutics.

[22] Lazaridou A, Kritikopoulou K, Biliaderis CG (2015). Barley β-glucan cryogels as encapsulation carriers of proteins: Impact of molecular size on thermo-mechanical and release properties. Bioactive Carbohydrates and dietary fibre.

[23] Sweeney T (2012). Effect of purified β-glucans derived from Laminaria digitata, Laminaria hyperborea and Saccharomyces cerevisiae on piglet performance, selected bacterial populations, volatile fatty acids and pro-inflammatory cytokines in the gastrointestinal tract of pigs. British Journal of Nutrition.

[24] NRC, N. R. C. Nutrient Requirements of Swine. National Academies Press, Washington, D.C. (2012).

[25] Pierce KM, Callan JJ, McCarthy P, O’Doherty JV (2005). Performance of weanling pigs offered low or high lactose diets supplemented with avilamycin or inulin. Animal Science.

[26] Lee C, Kim J, Shin SG, Hwang S (2006). Absolute and relative QPCR quantification of plasmid copy number in Escherichia coli. Journal of biotechnology.

[27] Vandesompele, J. *et al*. Accurate normalization of real-time quantitative RT-PCR data by geometric averaging of multiple internal control genes. *Genome biology*, **3**(7), research0034-1 (2002). 10.1186/gb-2002-3-7-research0034 PMC126239 12184808

[28] Van Soest PV, Robertson JB, Lewis BA (1991). Methods for dietary fiber, neutral detergent fiber, and nonstarch polysaccharides in relation to animal nutrition. Journal of dairy science.

[29] Littell, R. C. *SAS System for Mixed Models*. John Wiley & Sons, Ltd (2006).

[30] Pluske JR, Hampson DJ, Williams IH (1997). Factors influencing the structure and function of the small intestine in the weaned pig: a review. Livestock Science.

[31] EFSA Panel on Dietetic Products, Nutrition and Allergies (NDA). Guidance on the scientific requirements for health claims related to the immune system, the gastrointestinal tract and defence against pathogenic microorganisms. EFSA Journal, 14(1), 4369 (2016).

[32] O’Doherty JV, Dillon S, Figat S, Callan JJ, Sweeney T (2010). The effects of lactose inclusion and seaweed extract derived from Laminaria spp. on performance, digestibility of diet components and microbial populations in newly weaned pigs. Animal feed science and technology.

[33] Prohászka L, Baron F (1980). The predisposing role of high dietary protein supplies in enteropathogenic E. coli infections of weaned pigs. Zoonoses and Public Health.

[34] Dong GZ, Pluske JR (2007). The low feed intake in newly-weaned pigs: problems and possible solutions. Asian-australasian journal of animal sciences.

[35] Sangild PT (2006). Glucagon-like peptide 2 stimulates intestinal nutrient absorption in parenterally fed newborn pigs. Journal of pediatric gastroenterology and nutrition.

[36] Schmitt CC (2017). Intestinal invalidation of the glucose transporter GLUT2 delays tissue distribution of glucose and reveals an unexpected role in gut homeostasis. Molecular metabolism.

[37] Boudry G (2014). A high-protein formula increases colonic peptide transporter 1 activity during neonatal life in low-birth-weight piglets and disturbs barrier function later in life. British Journal of Nutrition.

[38] Round JL, Mazmanian SK (2009). The gut microbiota shapes intestinal immune responses during health and disease. Nature Reviews Immunology.

[39] Sommer F, Bäckhed F (2013). The gut microbiota—masters of host development and physiology. Nature Reviews Microbiology.

[40] Pajarillo EAB, Chae JP, Balolong MP, Kim HB, Kang DK (2014). Assessment of fecal bacterial diversity among healthy piglets during the weaning transition. The Journal of general and applied microbiology.

[41] Zhao W (2015). The dynamic distribution of porcine microbiota across different ages and gastrointestinal tract segments. PloS one,.

[42] Wang J (2017). Fecal microbiota succession of piglets from birth to post-weaning by 454 pyrosequencing analysis. Transactions of Tianjin University.

[43] MacDonald TT, Monteleone I, Fantini MC, Monteleone G (2011). Regulation of homeostasis and inflammation in the intestine. Gastroenterology.

[44] Ozdemir O (2012). Any Benefit of Probiotics for Autoimmune Gastrointestinal Diseases?. Journal of Pediatric Sciences.

[45] Brown GD, Gordon S (2005). Immune recognition of fungal β‐glucans. Cellular microbiology.

[46] Liu MJ (2013). ZIP8 regulates host defense through zinc-mediated inhibition of NF-κB. Cell reports.

[47] Bouwhuis MA (2016). The effect of maternal and postweaning seaweed extract supplementation on gut health in pigs after weaning and response to enterotoxigenic Escherichia coli K88 challenge. Journal of Animal Science,.

[48] Saloň I, Hanuš J, Ulbrich P, Štěpánek F (2016). Suspension stability and diffusion properties of yeast glucan microparticles. Food and Bioproducts Processing.

[49] Sultana A (2017). Microencapsulation of flavors by spray drying using Saccharomyces cerevisiae. Journal of Food Engineering.

[50] Falco CY, Sotres J, Rascón A, Risbo J, Cárdenas M (2017). Design of a potentially prebiotic and responsive encapsulation material for probiotic bacteria based on chitosan and sulfated β-glucan. Journal of colloid and interface science.

[51] Pérez, J. M. & Sauvant, D. Tables of composition and nutritional value of feed materials (No. 636.085 Sa89t Ej. 1 021121). INRA (2004).

